# Posterior Wnts Have Distinct Roles in Specification and Patterning of the Planarian Posterior Region

**DOI:** 10.3390/ijms161125970

**Published:** 2015-11-05

**Authors:** Miquel Sureda-Gómez, Eudald Pascual-Carreras, Teresa Adell

**Affiliations:** Department of Genetics and Institute of Biomedicine, University of Barcelona, Barcelona E-08028, Catalonia, Spain; msureda6@gmail.com (M.S.-G.); eudald.pascual@gmail.com (E.P.-C.)

**Keywords:** patterning, identity specification, wnt signaling, planarians

## Abstract

The wnt signaling pathway is an intercellular communication mechanism essential in cell-fate specification, tissue patterning and regional-identity specification. A βcatenin-dependent signal specifies the AP (Anteroposterior) axis of planarians, both during regeneration of new tissues and during normal homeostasis. Accordingly, four *wnts* (posterior *wnts*) are expressed in a nested manner in central and posterior regions of planarians. We have analyzed the specific role of each posterior *wnt* and the possible cooperation between them in specifying and patterning planarian central and posterior regions. We show that each posterior *wnt* exerts a distinct role during re-specification and maintenance of the central and posterior planarian regions, and that the integration of the different wnt signals (βcatenin dependent and independent) underlies the patterning of the AP axis from the central region to the tip of the tail. Based on these findings and data from the literature, we propose a model for patterning the planarian AP axis.

## 1. Introduction

The wnt signaling pathway is an intercellular communication mechanism with essential roles in cell-fate specification, tissue patterning and specification of regional identity [1]. Wnts, the secreted elements of the pathway, interact with the membrane receptors Frizzleds and their co-receptors (LRP4/5, Ror, Ryk) to transduce different signals that branch mainly in three pathways: the canonical or βcatenin-dependent wnt signaling, and two non-canonical or βcatenin-independent signals, which regulate either JNK (c-Jun N-terminal kinase) or PKC (Protein kinase C) pathways [1,2,3]. The βcatenin-dependent pathway exerts its function by regulating the nuclear translocation of βcatenin, and is mainly involved in cell-fate specification. One of the most conserved roles of βcatenin-dependent Wnt signaling is the specification of the AP (Anteroposterior) axis, where βcatenin is required to confer posterior features in most developmental models studied [4]. βcatenin-independent pathways are mainly involved in the control of cell shape and movements [5,6].

Planarians are an ideal model for the study of cell-fate specification and patterning, since they are extremely plastic. They are bilateral animals with a complex cephalized nervous system and a three-branched gut which converges into a pharynx, which takes in food and expulses debris through a ventral mouth [7]. Planarians can regenerate any amputated part, even the head, in a few days, and they continuously remodel their tissues while they grow and shrink according to food availability and temperature. Those capabilities are due to the presence of a population of totipotent stem cells all around their body, the neoblasts, which are able to differentiate to any cell type [7,8,9]. Because of its astonishing regenerative abilities, planarians have been established as a unique model to understand stem cell biology and the molecular mechanisms underlying patterning and regional identity specification. Specifically, the function of the Wnt signaling pathway has been extensively studied in planarians [10,11,12,13,14,15,16,17,18,19]. Due to its plasticity, even in the adult stage, the phenotypes generated when silencing Wnt pathway elements had no precedent in the field of developmental biology and were extremely informative. RNAi experiments demonstrate that *Smed-βcatenin1* is essential to pattern the AP axis in planarians, since its inhibition generates anteriorized phenotypes ranging from “tailless” planarians to “two-headed” planarians and, most strikingly, “radial-like hypercephalized” planarians [12,20]. Moreover, the study of several elements of the pathway confirms this function, since inhibition of *APC* and *axin*, elements of the βcatenin destruction complex, lead to posteriorization of planarians [11,18]. Interestingly, the *Smed-βcatenin1-*dependent Wnt signal is required to specify AP identities both during planarian regeneration and during homeostasis [12,17,18].

Consistent with the role of the βcatenin-dependent Wnt signal in AP axial specification, 4 *wnts* are expressed in the posterior part of planarians in a nested manner, which we name in this study posterior *wnts* (*Smed-wnt1*, *Smed-wnt11-1*, *Smed-wnt11-2* and *Smed-wnt11-5*) [15,17,18,19]. Since planarians such as *S. mediterranea* typically measure at least 1–2 mm in length, the field is too large to be patterned by a single morphogen. It has therefore been proposed that cooperation between posterior *wnts* could be required to pattern the AP axis [20]. Out of the four posterior *wnts*, however, only *Smed-wnt1* and *Smed-wnt11-2* have been studied functionally. During regeneration of the tail, *Smed-wnt1* inhibition leads to “tailless” or “two-headed” planarians, and *Smed-wnt11-2* inhibition leads to “tailless” planarians [14,15,19]. Although those two *wnts* seem to be regulators of βcatenin activity, because its silencing produces an anteriorized phenotype, the strong anteriorization of planarians produced after *Smed-βcatenin1* silencing has never been phenocopied by the inhibition of any *wnt*. The purpose of the present study is to analyze the specific role of each posterior *wnt* and the possible cooperation among them both during regeneration and maintenance of the AP axis. Our data demonstrates that each posterior *wnt* exerts a distinct function during posterior regeneration, and that the inhibition of all of them generates a stronger anteriorization than the inhibition of any of them alone. During homeostasis, simultaneous silencing of the four posterior *wnts* also generates a stronger phenotype than silencing any *wnt* alone, although a shift of posterior to anterior identity is never achieved. We conclude that the integration of the different Wnt signals (βcatenin dependent and independent) underlies the patterning of the AP axis from the central region to the tip of the tail.

## 2. Results

### 2.1. Individual Posterior Wnts Exert Specific Roles during Posterior Regeneration

To study the role of each posterior *wnt* during posterior regeneration, we first analyzed their expression pattern by *in situ* hybridization. In agreement with previous reports, the four posterior *wnts* are found to be expressed in a graded manner along the AP axis in intact planarians (Figure S1A) [19]. *Smed-wnt1* expression is restricted to few cells in the posterior midline; *Smed-wnt11-1* and *Smed-wnt11-2* are expressed from the mouth to the tip of the tail, and *Smed-wnt11-1* also in the mouth itself; and *Smed-wnt11-5* is expressed from the pre-pharyngeal region to the tip of the tail. Interestingly, all of them are expressed as a gradient, higher in the most posterior tip. Moreover, posterior *wnts* are also expressed in a temporal manner during posterior regeneration, being *Smed-wnt1* the first one, expressed few hours after cutting (Figure S1B) [14,19], followed by *Smed-wnt11-1* and *Smed-wnt11-2*, which are detected 2 days after cutting (Figure S1B) [19]. *Smed-wnt11-5* is expressed at all regeneration stages, since its expression is not lost after cutting the tail but just re-patterned (Figure S1B) [19]. Those expression patterns suggest that each posterior *wnt* could exert a specific role during posterior specification and patterning, and that the cooperation between them could enable a correct and complete posterior pattern.

**Figure 1:**
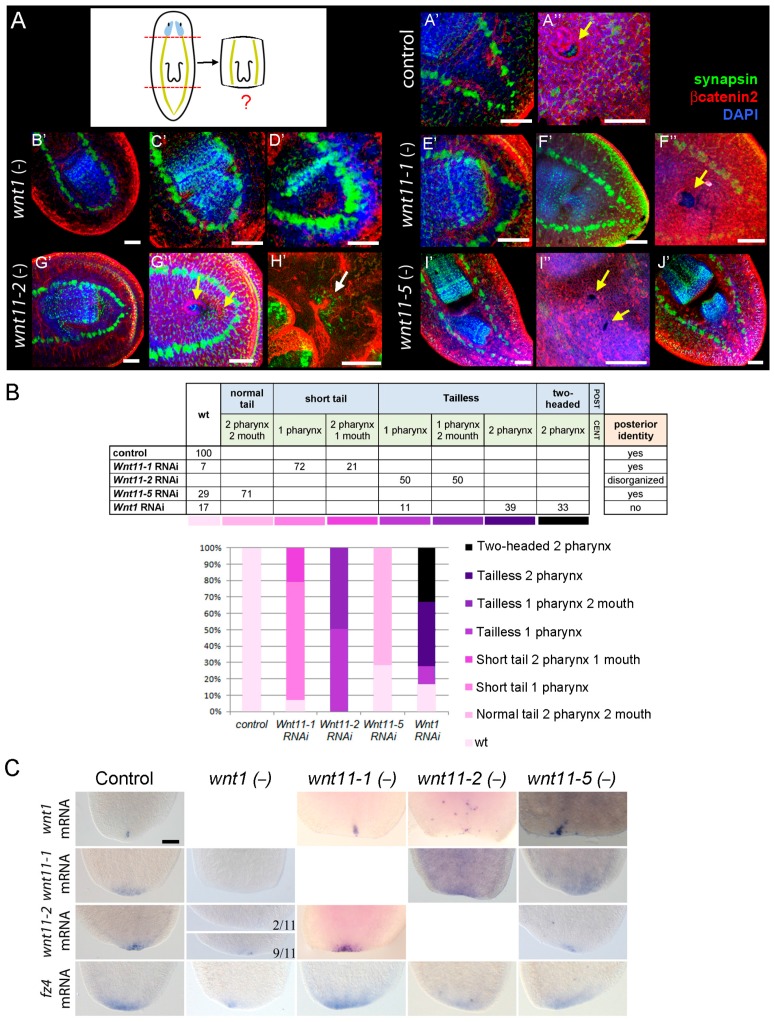
Each posterior *wnt* exerts a distinct function during planarian posterior regeneration. (**A**) Immunohistochemical analysis of planarian tail after silencing of *Smed-wnt1* (**B′**–**D′**), *Smed-wnt11-1* (**E**–**F′′**), *Smed-wnt11-2* (**G′**–**H′**) and *Smed-wnt11-5* (**I′**–**J′**). Anti-synapsin labels the nervous system (green), anti-βcatenin1 labels adherent junctions (red), and nuclei are stained with DAPI (4’,6-diamidino-2-phenylindole)(blue). **A′′**, **F′′**, **G′′** and **I′′** show a magnification of the plane corresponding to the mouth opening of **A′**, **F′**, **G′** and **I′**, respectively (mouth openings are indicated with yellow arrows). A primordium of a second pharynx in *Smed-wnt11-2* RNAi animals is shown in **H′** (white arrow). Animals were fixed at 20 days of regeneration. All images correspond to confocal z-projections; (**B**) Quantification of the different phenotypes observed after silencing each posterior *wnt*. (The number of animals analyzed for each condition was at least *n* = 14.). wt; wild type; (**C**) *In situ* hybridization analysis of the expression of posterior markers in 3-day regenerating posterior *wnt* RNAi. (The number of animals analyzed for each condition was at least *n* = 11.). Anterior is left/up, posterior is right/down in (**A**); anterior is up, posterior is down in **C**. Scale bar: 100 µm (**A**,**C**).

To test the specific role of each posterior *wnt*, we analyzed the morphology and pattern of the tail regenerated by planarians in which each posterior *wnt* alone was silenced. Phenotypes were analyzed by morphological observation and by immnohistochemistry with anti-synapsin and anti-βcatenin2 antibodies, to visualize the nervous and the digestive system, respectively (Figure 1 and Figure S2). As expected, inhibition of *Smed-wnt1* led to “tailless” and “two-headed” planarians (Figure 1A and Figure S1). Immunohistochemical analysis showed that “two-headed” planarians always differentiate a second pharynx in the opposite direction to the original one, according to the new axis generated in the posterior tip (Figure 1A(D′)). “Tailless” planarians showed a rounded closure of ventral nerve cords (VNCs) and an undefined posterior tip (Figure 1A(B′,C′)) [15]. Among “tailless” planarians two different phenotypes could be distinguished: animals in which only one pharynx was observed (sometimes in an opposite orientation) (Figure 1A(B′)) and animals in which two pharynges in opposite orientation could be observed (Figure 1A(C′)). Silencing of *Smed-wnt11-1* lead to the regeneration of shorter tails, in which the distance from the pharynx to the posterior tip was clearly shorter (Figure 1 and Figure S2). Immunohistochemical analysis showed that those animals close properly the VNCs in the posterior tip, and no signal of anteriorization can be observed (Figure 1A(E′)). Again, two different phenotypes could be distinguished when analyzing the central region, since in some animals a second pharynx appeared in parallel and very close to the pre-existing one (Figure 1A(F′)). Interestingly, two-pharynged *Smed-wnt11-1* RNAi animals never showed two mouths (Figure 1A(F′′)). Silencing of *Smed-wnt11-2* always lead to the regeneration of “tailless” planarians, as had been already reported (Figure 1 and Figure S1) [15,19]. Immunohistochemical analysis demonstrated that although *Smed-wnt11-2* RNAi animals only show the normal pre-existing pharynx, a second mouth appears in half of the animals (Figure 1A(G′,G′′)). A second pharynx associated to the second mouth has not been observed, although in few cases a pharynx primordium could be guessed (Figure 1A(H′)). *Smed-wnt11-5* RNAi animals apparently regenerated a perfect tail (Figure S2). Immunohistochemical analysis corroborates that VNCs close normally in the posterior tip. However, in most of the cases a second pharynx, oriented either in the same or in opposite direction with respect to the original one, can be observed (Figure 1A(I′,J′)). A second mouth always differentiates associated to the second pharynx (Figure 1A(I′′)). Thus, in *Smed-wnt11-5* RNAi animals, posterior identity appears normal but the central region appears duplicated. The quantification of the different phenotypes observed after silencing each posterior *wnt* alone allows the visualization of the different degrees of anteriorization generated (Figure 1B).

We then analyzed posterior identity specification of planarians in which posterior *wnts* were silenced. Posterior *wnts* and *fz4* were used as markers [18]. Results show that after *Smed-wnt1* RNAi the rest of posterior *wnts* and *fz4* disappear or decrease significantly, demonstrating the loss of posterior identity in “tailless” and “two-headed” phenotypes (Figure 1C). In contrast, after *Smed-wnt11-1* and *Smed-wnt11-5* RNAi, all posterior markers were expressed in the same pattern and levels as in controls, in agreement with the normal posterior closure of the VNCs in the posterior tip (Figure 1C). Thus, *Smed-wnt11-1* and *Smed-wnt11-5* RNAi animals have normal posterior identity. *Smed-wnt11-2* RNAi animals displayed a significant decrease in the expression of posterior markers, according to the “tailless” phenotype observed. Expression of *Smed-wnt1* appeared not only diminished but totally disorganized (Figure 1C) [19]. Interestingly, expression of *Smed-wnt11-1* and *Smed-wnt11-2* was found to be dependent on *Smed-wnt1*, although it remains unclear whether this is a direct regulation or a consequence of the loss of posterior identity.

Taken together, these results suggest that *Smed-wnt1* and *Smed-wnt11-2* specify posterior identity, although only *Smed-wnt1* RNAi animals exhibit a shift in polarity. Moreover, *Smed-wnt11-2* exerts a role in patterning or specifying central identity, since its inhibition duplicates the mouth. *Smed-wnt11-1* and *Smed-wnt11-5* are not required to specify the identity of the posterior tip. However, they have a role in patterning or specifying the central region, since ectopic pharynges differentiate when they are silenced. *Smed-wnt11-1* would be also required to properly elongate the tail.

### 2.2. Smed-wnt11-2 and Smed-wnt11-5 but not Smed-wnt11-1 Cooperate with Smed-wnt1 in Specifying Posterior Identity

**Figure 2:**
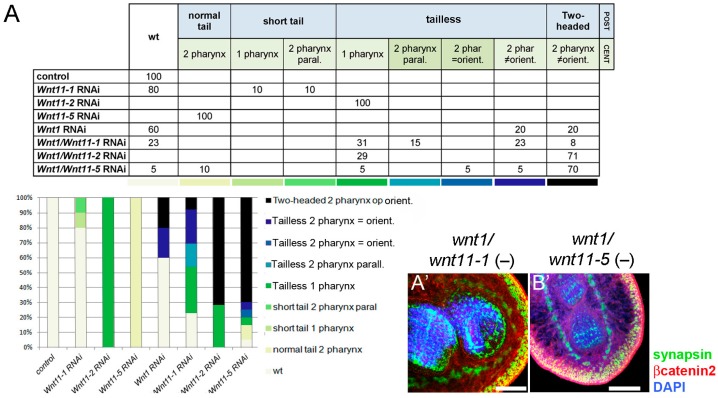

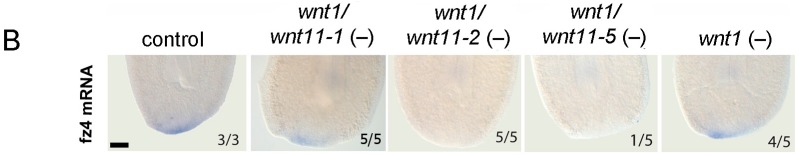
Cooperation of posterior *wnts* to specify posterior identity during regeneration. (**A**) Quantification of the different phenotypes observed after silencing each posterior *wnt* alone and *Smed-wnt1* in combination with the other posterior *wnts*. Two new phenotypes appeared after silencing *Smed-wnt1/Smed-wnt11-1* (**A′**) and *Smed-wnt1/Smed-wnt11-5* (**B′**), both of which show a “tailless” morphology next to the differentiation of a second pharynx alongside the original one. Animals were fixed at 20 days of regeneration. (The number of animals analyzed for single RNAi was *n* = 4–10 and for double *n* = 7–20.). **A′**,**B′** images correspond to confocal z-projections; and (**B**) *In situ* hybridization analysis of the expression of posterior markers in 3-day regenerating animals in which *Smed-wnt1* was silenced together with the other posterior *wnts*. (The number of animals analyzed for each condition was *n* = 3–5.). Anterior is left/up, posterior is right/down in **A′**,**B′**; anterior is up, posterior is down in (**B**). Scale bar: 100 µm (**A**,**B**).

To study whether posterior *wnts* play a cooperative role in posterior specification and patterning, we silenced *Smed-wnt1* (the only *wnt* that leads to shift of posterior to anterior identity upon silencing) simultaneously with *Smed-wnt11-1*, *Smed-wnt11-2* or *Smed-wnt11-5*. The resulting phenotypes were analyzed by immunohistochemistry with anti-synapsin and anti-βcatenin2 antibodies to visualize the nervous and digestive systems (Figure 2A). The phenotypes obtained after double inhibition were quantified and compared with those obtained after single inhibition of each posterior *wnt* (Figure 2A), allowing visualization of the degree of cooperation between the different posterior *wnts* in central and posterior specification. In these experiments, the penetrance of the phenotypes of single *wnt* RNAi was milder than in the experiments shown above, since half the amount of dsRNA was injected for each gene in order to maintain the total amount of dsRNA injected per animal (see Section 4.2). Quantification of the different phenotypes shows that simultaneous silencing of *Smed-wnt1* together with *Smed-wnt11-2* or *Smed-wnt11-5* increased the number of “two-headed” planarians from 20% in *Smed-wnt1* RNAi planarians to 70% in the doubles [14]. In contrast, simultaneous silencing of *Smed-wnt1* together with *Smed-wnt11-1* decreased the frequency of “two-headed” planarians from 20% to 8%. Interestingly, two new phenotypes not observed in the single inhibition experiment appeared in these experiments. Firstly, we observed “tailless” planarians with two pharynges in parallel, which is the addition of the suppression of the posterior identity after *Smed-wnt1* silencing together with the appearance of an ectopic pharynx after *Smed-wnt11-1*. In addition, “tailless” planarians were observed with two pharynges in tandem and in the same orientation, which is the addition of the suppression of the posterior identity after *Smed-wnt1* silencing and the duplication of the central identity produced by *Smed-wnt11-5* silencing (Figure 2A(A′,B′)). According to the phenotypes observed, analysis of the posterior marker *fz4* in the double RNAi planarians revealed a loss or reduction in *Smed-wnt1/Smed-wnt11-2* and *Smed-wnt1/Smed-wnt11-5* RNAi planarians (Figure 2B). *Smed-wnt1/Smed-wnt11-1* RNAi animals also displayed a mild reduction of *fz4* expression, possibly due to the inhibition of *Smed-wnt1*. Taken together, these results demonstrate that *Smed-wnt11-2* and *Smed-wnt11-5*, but not *Smed-wnt11-1*, cooperate with *Smed-wnt1* in specifying posterior identity. The contribution of *Smed-wnt11-2* in posterior specification could be predicted according to its requirement in single RNAi experiments. However, the contribution of *Smed-wnt11-5* in posterior specification should be in cooperation with *Smed-wnt1*, since its inhibition alone never induces posterior defects. The possible cooperation between *Smed-wnt11-1*, *Smed-wnt11-2* and *Smed-wnt11-5* in the specification and patterning of the central region requires further attention.

### 2.3. Silencing of All Posterior Wnts Together Is Insufficient to Transform Posterior Identity into Anterior during Homeostasis

**Figure 3:**
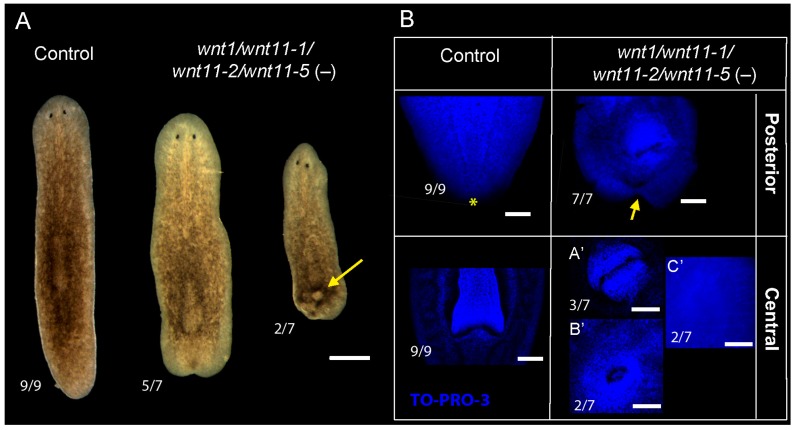

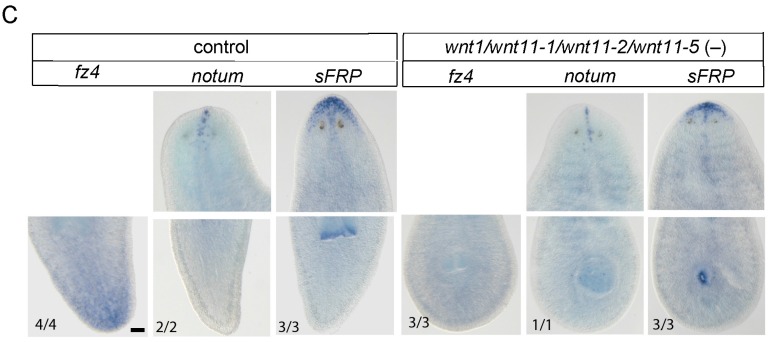
Silencing of all posterior *wnts* during homeostasis generates a strong “tailless” phenotype, without neither posterior nor anterior identity (**A**) After 6 rounds of *Smed-wnt1*/*Smed-wnt11-1*/*Smed-wnt11-2*/*Smed-wnt11-5* inhibition, planarians show a “tailless” phenotype in which the central region is also affected, since the pharynx cannot be maintained (yellow arrow points to a hole generated after the expulsion of the pharynx). (The number of animals analyzed for each condition was *n* = 7–9); (**B**) TO-PRO-3 staining of the nucleus shows the “tailless” shape of RNAi planarians (yellow arrow) compared to controls (yellow asterisk), and the disorganization of the central region (**A′**, two pharynges; **B′**, disorganized pharynx; **C′**, no pharynx, after expulsion). All images correspond to confocal z-projections; and (**C**) “Tailless” *Smed-wnt1*/*wnt11-1*/*wnt11-2*/*wnt11-5* RNAi animals do not show expression of either posterior (*Fz4*) or anterior (*sFRP*, *notum*) markers in the posterior region. Anterior markers are normally expressed in the anterior region. Anterior is up, posterior is down in **all images**. Scale bar: 500 µm (**A**), 100 µm (**B**, **A′**, **B′** and **C′**) and 100 µm (**C**).

βcatenin-dependent Wnt signaling is also required for the maintenance of posterior identity and pattern during planarian homeostasis, since *Smed-βcatenin1* inhibition in intact planarians produces the appearance of ectopic eyes and brain in the posterior tip [12,17,18]. To analyze the possible cooperation between posterior *wnts* in the maintenance and pattern of the AP axis during homeostasis, we silenced them simultaneously and analyzed the resulting phenotypes after 6 weeks of inhibition. As a previous step, we silenced each *wnt* alone and showed that posterior eyes were not induced in any case (Figure S3). However, *Smed-wnt11-1* and *Smed-wnt11-2* RNAi planarians did show “tailless” phenotypes. RNAi of the four posterior *wnts* simultaneously produced an evident reduction of the tail, generating a strong “tailless” phenotype. In those animals, the pattern of the central region was also affected, and 3 types of central phenotypes could be distinguished: animals with two pharynges in opposite orientation, animals with a disorganized pharynx, and animals without a pharynx, due to its expulsion (Figure 3A,B). Despite the strong phenotype observed in posterior *wnt* RNAi planarians, the differentiation of ectopic anterior structures never occurred. The analysis of anterior and posterior identity markers corroborates the “tailless” phenotype, since RNAi planarians completely lost the expression of the posterior marker *fz4*, and the anterior markers *sFRP* [18] and *notum* [16] never appear in the posterior region (Figure 3C). Moreover, *sFRP* staining also revealed disorganization of the pharynx (Figure 3C).

Taken together, these results show that disruption of the central and posterior regions in intact planarians is much stronger when silencing all posterior *wnts* simultaneously than when they are silenced individually, providing evidence of cooperation in the patterning of these regions. However, in contrast to the results reported for *Smed-βcatenin1* silencing [12], a shift of posterior identity to anterior was not observed under homeostatic conditions.

## 3. Discussion

**Figure 4:**
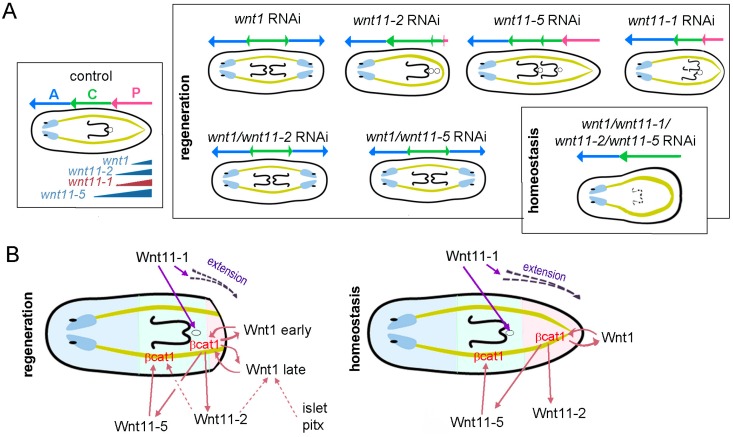
Summary and working model. (**A**) Scheme of the phenotypes generated after silencing the different posterior *wnts* (blue A, anterior; green C, central; pink P, posterior). The strongest phenotype is represented; (**B**) Proposed model of the function of posterior *wnts* in central and posterior specification and patterning (in the planarian: blue is anterior; green is central and pink is posterior).

Depending on the dose and time of inhibition, *Smed-βcatenin1* RNAi induces a gradual anteriorization of planarians, from “tailless” to “radial-like hypercephalized” animals. Consequently, it has been proposed that the graded activation of Smed-βcatenin1 from posterior to anterior is responsible for specifying the whole AP axis in planarians [12,20]. However, the *wnts* responsible for the nuclear localization of Smed-βcatenin1 in such a broad domain remained mainly elusive. Until now, only the involvement of *Smed-wnt1* in Smed-βcatenin1 nuclearization had been suggested, since it is the only *wnt* for which inhibition induces the appearance of a posterior head during posterior regeneration [14,15]. However, the strong anteriorization observed after *Smed-βcatenin1* silencing has never been observed following inhibition of any *wnt*. In this study, we analyzed the function of the four *wnts* which are expressed in the posterior part of planarians (posterior *wnts*) and explored the possibility that they cooperate to pattern planarian AP axis (Figure 4). Our results confirm that *Smed-wnt1* is the only *wnt* for which inhibition leads to a shift in posterior polarity during regeneration, when posterior identity must be re-specified. Moreover, we reproduce the “tailless” phenotypes after inhibition of *Smed-wnt11-2* [15,19], which also must exert a role in posterior specification, according to the decreased and disorganized pattern of posterior markers. In contrast, our results demonstrate that *Smed-wnt11-1* and *Smed-wnt11-5* are not required for posterior specification, since the tip of the tail in those RNAi animals regenerates normally and posterior markers are normally expressed. Interestingly, our data point to a role for *Smed-wnt11-5* in the specification or patterning central identity, since *Smed-wnt11-5* RNAi animals regenerate a second pharynx and mouth posteriorly to the pre-existing one. The shorter tail of *Smed-wnt11-1* RNAi planarians could indicate a role for this *wnt* in the extension of the tail. Moreover, our data suggests that *Smed-wnt11-1* could exert a direct role in the formation of the mouth, since it is expressed in this organ, and *Smed-wnt11-1* RNAi planarians never duplicate the mouth despite the presence of two pharynges.

Based on the results obtained in this study, we hypothesize that *Smed-wnt1*, *Smed-wnt11-2* and *Smed-wnt11-5* could act in a *βcatenin*-dependent manner, nuclearizing Smed-βcatenin1 in different domains along the AP axis (Figure 4B). Whereas *Smed-wnt1* and *Smed-wnt11-5* could be direct regulators of the βcatenin destruction complex in the posterior and central region, respectively, *Smed-wnt11-2* could be modulating Smed-βcatenin1 activity indirectly, at least in the posterior region. This possibility is supported by the observation that *Smed-wnt1* does not disappear but shows a disorganized pattern in *Smed-wnt11-2* RNAi planarians (Figure 4B). At this point, it should be noted that two different stages of *Smed-wnt1* expression occur during regeneration: an early *Smed-wnt1* expression, which occurs during wounding and is stem-cell independent, and a late *Smed-wnt1* expression, localized in the most posterior tip (the area which would correspond to the posterior organizer), that is stem-cell dependent [19]. We hypothesize that posterior identity is established by early *Smed-wnt1* expression, which triggers the sustained activation of *Smed*-βcatenin1 in posterior regions through the subsequent activation of the late *Smed-wnt1* expression (Figure 4B). *Smed-wnt11-2*, for which inhibition leads to “tailless” planarians, would be required for the proper expression pattern of the late *Smed-wnt1*. The concentration of *Smed-wnt1* in the posterior tip would be essential for the establishment of the organizing region, which is responsible for growth and pattern rather than for identity specification. Additional factors, such as *Smed-pitx* or *Smed-islet*, could cooperate with *Smed-wnt11-2*, since their inhibition leads to suppression of late *Smed-wnt1* expression and regeneration of “tailless” planarians [21,22]. It has been proposed that the “tailless” phenotype could also be the result of *Smed-wnt11-2* acting in the establishment of the posterior midline [19]. In our view, the abolishment of the posterior midline goes together with the disruption of the posterior organizer. *Smed-wnt11-1* RNAi animals regenerate a shorter tail showing a proper terminal identity. Moreover, their occasionally duplicated pharynx never locates in tandem, like in *Smed-wnt11-5* or *Smed-wnt1* RNAi planarians. For that reason, we hypothesize that *Smed-wnt11-1* would not function in a *βcatenin*-dependent manner but it would be involved in the non-canonical/βcatenin-independent Wnt signaling, a well known mechanism to regulate migration and cell movement, which are the main morphogenetic processes required for tissue extension and epithelial rearrangements [6]. The possible non-canonical function of *Smed-wnt11-1* and *Smed-wnt11-2* compared to the βcatenin-dependent function of *Smed-wnt11-5* is further supported by their evolutionary origin, since phylogenetic analysis shows that *Smed-wnt11-5* does not branch with Wnt11 but with the Wnt4 family [23]. Moreover, a *wnt4* has been suggested to act in a βcatenin-dependent manner in the platyhelminth *Schistosoma* [24]. Altogether, our results suggest that posterior *wnts* act in cooperation to provide a precise spatiotemporal control of the AP axis, from the pre-pharyngeal region to the tip of the tail.

The cooperation and integration of βcatenin-dependent and -independent Wnt signaling has been demonstrated to be essential also in the patterning of the AP neuroectoderm axis in sea urchin [25]. In cnidarians, it has been suggested that the patterning of the oral-aboral axis could be established by the cooperation between different Wnts, a “Wnt code”, which would exert the function of the Hox code in bilatelians [26]. If the cooperation of posterior *wnts* is also required for maintenance of the AP pattern during homeostasis in planarians, then we expect that inhibition of the whole posterior *wnt* complement would lead to the abolishment of the identities from the pre-pharynx to the tail. Our results show that inhibition of posterior *wnts* during homeostasis one by one never induces the appearance of ectopic anterior structures but only generates mild “tailless” phenotypes. In contrast, inhibition of all posterior *wnts* together leads to a strong “tailless” phenotype, in which posterior markers disappear and also the central region is affected, since the pharynx cannot be maintained, which in fact is a feature of *Smed-βcatenin1* RNAi animals. This result confirms the hypothesis that posterior *wnts* cooperate to pattern the AP axis, including central and posterior regions. However, inhibition of the whole posterior *wnt* complement never induces the appearance of ectopic anterior structures, as occurs after *Smed-βcatenin1* silencing. One reason could be that silencing all posterior *wnts* simultaneously affects not only the bcatenin-dependent but also the bcatenin-independent Wnt signaling, which could prevent cell tip specification. Further RNAi analysis with different combinations of posterior *wnts* should be performed. A second reason could be that RNAi inhibition of the secreted elements of the pathway is less efficient than inhibition of the intracellular element, particularly considering that we are silencing four genes simultaneously. However, it must be noted that silencing of *Smed-wnt1* alone produces a strong anteriorization of planarians during regeneration but has no apparent phenotype during homeostasis. This observation could indicate that the signals which trigger posterior identity are different in the context of regeneration, when the posterior organizer must be re-specified, compared with the context of homeostasis, when the posterior organizer must be only maintained. A robust signaling network could underlie the maintenance of the posterior organizer (high levels of Smed-βcatenin1). Only the inhibition of *Smed-βcatenin1* itself or downstream elements, like *Smed-teashirt* [27], or the removal of the organizer after a posterior amputation, enables its re-specification towards a different fate.

## 4. Experimental Section

### 4.1. Planarian Culture

Planarians used in the presented experiments correspond to the clonal strain of *S. mediterranea* known as BCN-10 biotype. They were maintained as previously described [28]. Planarians used in these experiments were 4–6 mm length and were starved for 1 week before used for experiments.

### 4.2. RNAi Analysis

Double-stranded RNAs (dsRNAs) used in these experiments were synthesized by *in vitro* transcription (Roche) as previously described [29]. dsRNA microinjections were performed in the digestive system of planarians following the standard protocol of a 3 × 32 nL/injection of double-stranded (ds) RNA for three consecutive days before being amputated [29]. In regeneration experiments, 2 consecutive rounds of dsRNA injections were performed (1 round corresponds to 1 week, in which animals are injected on the first 3 days and amputated on the fourth). Animals were amputated transversally in 3 parts (heads, trunks and tails). In homeostasis experiments, 1 round of injection corresponds to 1 week in which dsRNA is injected on the first 3 days. Control animals were injected with dsRNA for the green fluorescent protein (GFP) sequence. In simultaneous gene-silencing experiments, the total amount of dsRNA injected for each gene and also the total amount of dsRNA injected in each animal was maintained constant by injecting the amount of GFP required.

### 4.3. Whole-Mount in Situ Hybridization

The RNA probes used in the present experiments were synthesized *in vitro* using Sp6 or T7 polymerase (Roche, Sant Cugat del Vallès, CAT, Spain) and DIG-modified ribonucleotides (Roche). Afterwards they were purified by ethanol precipitation and 7.5 M ammonium acetate addition. For *in situ* hybridization, animals were killed with HCl 2%, and fixed in Carnoy. An *in situ* Pro hybridization robot (Abimed/Intavis, Tübinguen, BW, Germany) was used for the *in situ* protocol, as previously described [30,31]. The temperature used for hybridizations was 56 °C, and were carried out for 16 h. A Leica MZ16F microscope (Leica Microsystems, Mannhiem, BW, Germany) was used to observe the samples. Images were captured with a ProgRes C3 camera from Jenoptik (Jena, TH, Germany).

### 4.4. Immunostaining

Immunostaining was carried out as described in previous studies [32]. The antibodies used in these experiments were: anti-synapsin (anti-SYNORF1,1:50, Developmental Studies Hybridoma Bank, Iowa City, IA, USA), anti-Smed-β-catenin2 (1:1000) [33] and anti-α-tubulin (AA4, 1:20, Developmental Studies Hybridoma Bank). Alexa 488-conjugated goat anti-mouse (1:400, Molecular Probes, Waltham, MA, USA) and Alexa 568-conjugated goat anti-rabbit (1:1000, Molecular Probes) were used as a secondary antibodies. Nuclei were stained with DAPI (1:5000) or TO-PRO^®^-3 (1:3000, Thermo Fisher Scientific, Waltham, MA, USA). A Leica TCS-SP2 (Leica Lasertechnik, Heidelberg, BW, Germany) adapted for an inverted microscope (Leitz DMIRB, Leica Lasertechnik, Heidelberg, BW, Germany) and a Leica TCS SPE (Leica Microsystems, Mannhiem, BW, Germany) were used to obtain confocal images. Representative confocal stacks for each experimental condition are shown.

## Supplementary Materials

Supplementary materials can be found at http://www.mdpi.com/1422-0067/16/11/25970/s1.
