# Supplementary material for: Posterior Wnts Have Distinct Roles in Specification and Patterning of the Planarian Posterior Region
Source: Int J Mol Sci. 2015 Nov 5;16(11):26543–54. doi: 10.3390/ijms161125970 (PMC4661829; doi:10.3390/ijms161125970)
Supplement: Supplementary file 1 [file ijms-16-25970-s001.pdf]

International Journal of
Molecular Sciences

MDPI

Supplementary Information

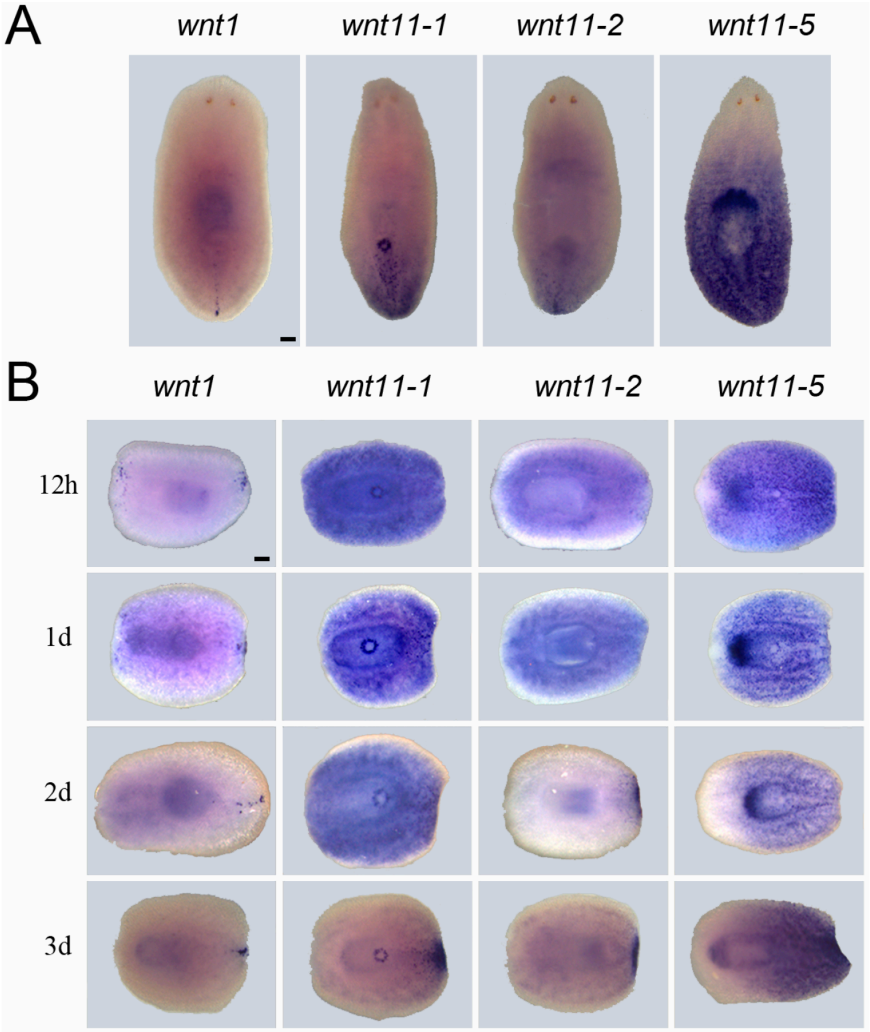

Figure S1. Expression pattern of posterior *wnts* in intact and regenerating animals. (**A**) *In situ* hybridization of posterior *wnts* in intact animals. *Smed-wnt1* is expressed as a stripe of cells in the posterior dorsal midline; *Smed-wnt11-1* is expressed in the mouth and as a posterior gradient from the mouth to the tail; *Smed-wnt11-2* is expressed as a posterior gradient, concentrated in the posterior midline; and *Smed-wnt11-5* is expressed in the esophagus and as a gradient from the prepharynx to the tail; and (**B**) *In situ* hybridization of posterior *wnts* in regenerating trunks at 12 h, 1 day, 2 days and 3 days post-amputation. Anterior blastemas are shown on the left and posterior blastemas on the right. The first wnt to be expressed in the regenerating region is *Smed-wnt1*, at 12 h. At 1 day, *Smed-wnt1* decreases its expression in anterior blastemas and concentrates in the posterior. At 2 days, it recovers the expression pattern observed in intact animals (posterior dorsal midline) and disappears in anterior blastemas. *Smed-wnt11-1* expression is only maintained in the mouth during early regeneration stages, and appears in the regenerating region at day 2, at the same time that *Smed-wnt11-2*. *Smed-wnt11-5* keeps the expression observed in intact animals. At 2 days of regeneration, it starts to re-scale from anterior to posterior to recover the gradient seen in intact animals. At 3 days of regeneration, the expression of all posterior *wnts* resembles the one observed in intact planarians. (The number of animals analyzed for each condition was at least $n = 5$.) Scale bar: 100 µm (**A**,**B**).

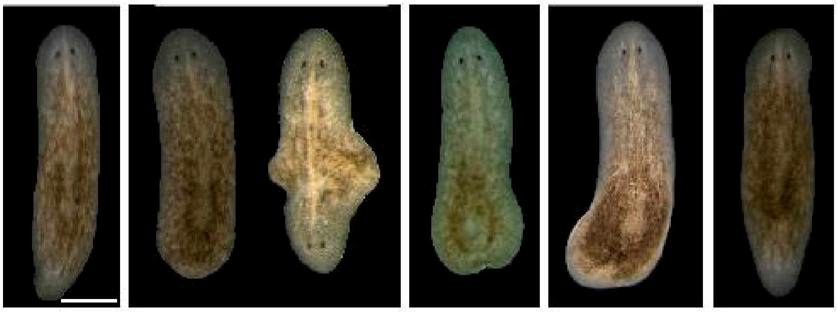

Figure S2. Phenotypes of posterior *wnts* RNAi during regeneration. Stereomicroscope views of regenerating trunk pieces showing the different phenotypes. From left to right: control animals have a wild-type appearance; *Smed-wnt1* RNAi planarians exhibit 2 different phenotypes, "tailless" (50% of animals) and "two-headed" (33% of animals); *Smed-wnt11-1* RNAi generates "short tail" planarians (93% of animals); *Smed-wnt11-2* RNAi generate "tailless" planarians (100% of animals); and *Smed-wnt11-5* RNAi planarians have a wild type tail. Images correspond to 20 days regenerating animals. (The number of animals analyzed for each condition was at least $n = 14$.) Scale bar: 500 µm.

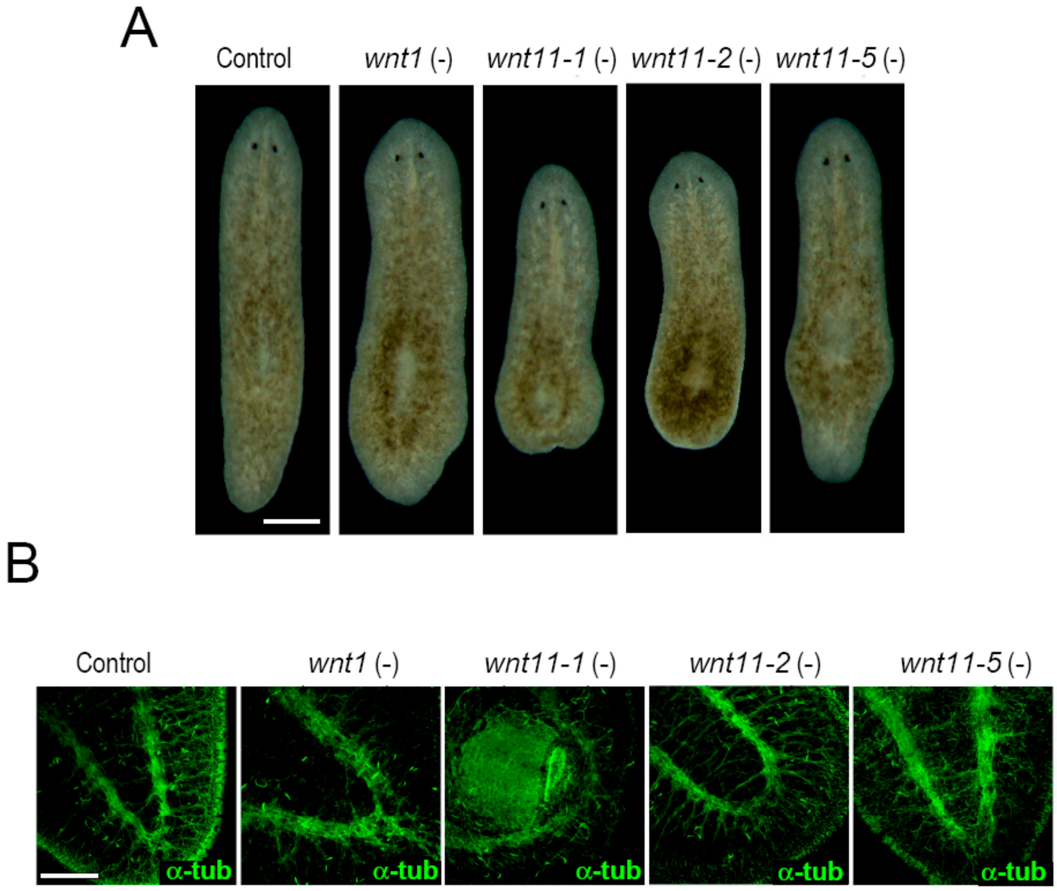

Figure S3. Phenotypes of posterior *wnt* RNAi during homeostasis. (**A**) Stereomicroscope view of the different phenotypes following RNAi in intact planarians. From left to right: controls resemble wild type; *Smed-wnt1* RNAi planarians also resemble wild type; *Smed-wnt11-1* RNAi generates "short tail" planarians; *Smed-wnt11-2* RNAi generates "tailless" planarians (100% of animals); and *Smed-wnt11-5* RNAi planarians have a tail that resembles wild type. (The number of animals analyzed for each condition was at least $n = 10$.); and (**B**) α-Tubulin immunostaining showing the morphology of the ventral nerve cords in the posterior tip of planarians after silencing posterior *wnts*. All images correspond to 20 days regenerating animals. Scale bar: 500 µm (**A**) and 100 µm (**B**).
